# Management of Arrhythmic Mitral Valve Prolapse: Potential Impact and Current Evidence

**DOI:** 10.31083/RCM38956

**Published:** 2025-09-19

**Authors:** Serghei Covantsev, Andia Taghdiri, Anna Bumbu, Natalia Pichugina, Anna Sukhotko

**Affiliations:** ^1^Department of Clinical Research and Development, Botkin Hospital, 125284 Moscow, Russia; ^2^Faculty of Medicine, Ivane Javakhishvili Tbilisi State University, 0159 Tbilisi, Georgia; ^3^Department of Ultrasonography, Botkin Hospital, 125284 Moscow, Russia; ^4^Department Clinical Research and Development, Botkin Hospital, 125284 Moscow, Russia

**Keywords:** cardiology, mitral valve, mitral valve surgery, arrhythmia

## Abstract

Mitral valve prolapse (MVP), also known as floppy mitral valve syndrome, systolic click-murmur syndrome, and billowing mitral leaflets, is a developmental anomaly caused when one or two abnormal valve leaflets are displaced into the left atrium below the mitral valve annulus during systole. MVP is observed in 2–3% of patients in the general population and is the leading cause of mitral regurgitation (MR) in developed countries. Overall, MVP is considered a benign developmental anomaly; however, evidence suggests that MVP is associated with sudden cardiac death. Thus, there have been ongoing discussions about the optimal management of this patient group, which includes both pharmacological treatment and surgical interventions. This review aimed to provide an overview of the benign and arrhythmic MVP (AMVP), its diagnostic options, and management possibilities.

## 1. Introduction

Mitral valve prolapse (MVP), also known as floppy mitral valve syndrome, systolic click-murmur syndrome, and billowing mitral leaflets, is a developmental anomaly caused when one or two abnormal valve leaflets are displaced into the left atrium below the mitral valve annulus during systole [1, 2]. MVP is observed in 2–3% of patients in the general population and is the leading cause of mitral regurgitation (MR) in developed countries [3, 4]. Overall, MVP is considered a benign developmental anomaly; however, evidence from the Framingham study suggests that MVP is seen in 2.4% of patients with sudden cardiac death (SCD) [3]. This association leads to an alarming suspicion that the lifelong course of MVP is not always benign. Several articles since the 1980s have reported sudden cardiac death in young asymptomatic patients with MVP [5]. Meanwhile, it is well known that arrhythmogenesis is a complex process involving several pathological mechanisms that alter the electrical characteristics of the heart [6, 7].

A particular type of MVP is sometimes referred to as “Barlow disease”, which was first reported by Barlow *et al*. in the 1960s, before the era of echocardiography [2]. This particular type of malignant phenotype is characterized by thickened, redundant leaflets, bileaflet prolapse, and elongated chordae, with or without mitral annular disjunction (MAD) [4, 8]. One possible reason is myocardial fibrosis at the level of the papillary muscles and the infero-basal left ventricular (LV) wall, which, in turn, leads to contraction abnormalities [9, 10, 11]. There have been ongoing discussions about the optimal management of this patient group, which includes both pharmacological treatment and surgical interventions.

Therefore, the current review aims to provide an overview of the benign and arrhythmic MVP (AMVP), its diagnostic options, and management possibilities.

## 2. Literature Review

### 2.1 Arrhythmic Mitral Valve Prolapse

**Fig. 1.**
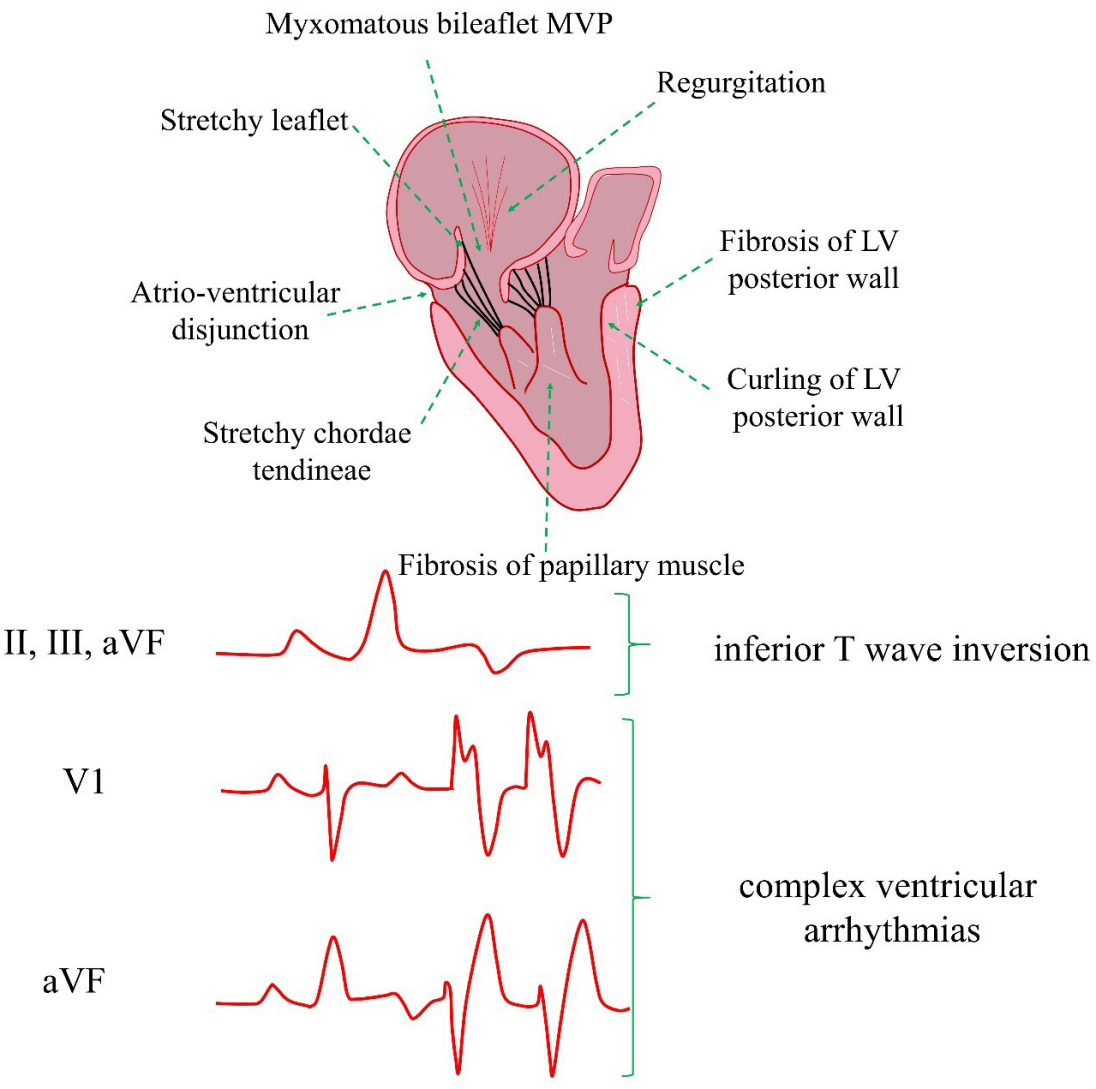
**AMVP cardiac risk factors and pathogenesis**. AMVP, arrhythmic mitral valve prolapse; MVP, mitral valve prolapse; LV, left ventricular; VF, ventricular fibrillation.

Understanding of the true mechanism of arrhythmias in MVP remains limited. However, it appears that myocardial fibrosis in the sub-valvular apparatus represents the main anatomical area of interest. Subsequently, this process can trigger electrical activity due to the mechanical stretch of the papillary muscles. Meanwhile, endocardial and myocardial fibrotic changes in the papillary muscles and left ventricle can also lead to abnormal electrical activity [12]. Moreover, several pathological changes can be encountered in AMVP (Fig. 1) [9, 10, 11].

However, current guidelines define AMVP as “a combination of MVP (with or without MAD), with frequent and/or complex ventricular arrhythmia (VA), in the absence of any other well-defined arrhythmic substrate (e.g., primary cardiomyopathy, channelopathy, active ischemia, or ventricular scar due to another defined etiology), regardless of MR severity” [12].

The two main phenotypes include AMVP due to severe degenerative mitral regurgitation and AMVP with severe myxomatous disease irrespective of degenerative mitral regurgitation (Table 1, Ref. [13, 14, 15, 16, 17, 18]).

**Table 1.** **Comparison of the AMVP phenotypes**.

Criteria	AMVP due to severe degenerative mitral regurgitation	AMVP with severe myxomatous disease, irrespective of degenerative mitral regurgitation	References
Excess mortality	High-risk of excess mortality	Equivalent to the general population	[13, 14, 15]
Risk factors of mortality	Atrial arrhythmias, reduced LV systolic function, and severe heart failure symptoms	In total, 9% are present with high-risk arrhythmias	[13, 16, 17]
		Patients with presyncope or syncope	
		Patients with inferior ST-T changes and premature ventricular complexes	
MVP	MVP with at least moderate to severe MR yields excess mortality	Prone to develop arrhythmia over time, dependent on the degree of MV degeneration	[13, 16]
Surgery	Surgical MR correction tends to reduce ventricular arrhythmic events, SCD rate, and overall mortality	A small subset may require surgery, but most likely this group of patients requires repeated/extended cardiac monitoring	[13, 14, 16, 18]

MR, mitral regurgitation; SCD, sudden cardiac death.

Despite data from cardiac magnetic resonance imaging (MRI) studies demonstrating fibrosis near the mitral annulus, the current theory suggests that the primary mechanism of VA leading to SCD is non-reentrant; however, numerous LV contraction abnormalities (Pickelhaube sign and LV mechanical dispersion) also exist [19, 20, 21]. Myocardial fibrosis detected by MRI was found to be strongly associated with the arrhythmic phenotype [17]. However, the AMVP phenotype seems to affect a small subset of patients with MVP.

Essayagh and coworkers [22] evaluated a cohort of 595 patients with MVP who underwent clinical examination, Holter monitoring for 24 h, and Doppler echocardiographic characterization. Essayagh and coworkers [22] detected the presence of VA in 43% of patients (moderate ventricular tachycardia in 27% of cases, and severe in 9%). VA was associated with male sex, bileaflet prolapse, marked leaflet redundancy, mitral annulus disjunction, a larger left atrium and LV end-systolic diameter, and T-wave inversion (TWI)/ST-segment depression (*p* ≤ 0.001) [22]. The results of the study were further reinforced by a meta-analysis of nine studies from 1985 to 2023, which included 2279 patients and demonstrated that bileaflet prolapse, TWI, MAD, late gadolinium enhancement, and a history of syncope could define high-risk phenotypes in patients with MVP [23].

Several other meta-analyses have presented similar results, including additional risk factors such as a longer anterior mitral leaflet, a posterior mitral leaflet, a thicker anterior mitral leaflet, and a longer mitral annulus disjunction compared to patients without arrhythmia [24, 25].

### 2.2 Imaging and Anatomical Changes

#### 2.2.1 Echocardiography and Anatomical Changes

MVP can be defined as the displacement of one or both MVs during systole. The characteristic echocardiography picture is displacement upwards by at least 2 mm above the level of the mitral annulus in the sagittal view. Echocardiography enables the diagnosis of MVP and the two main underlying phenotypes: myxomatous MVP and fibroelastic deficiency [12, 26].

Myxomatous MVP is characterized by an excess of tissue, thickening, and/or elongation of the chordae, with or without annular dilation and calcification. MVP with fibroelastic deficiency is more common and presents itself by thinning and elongation of the chordae [12, 26]. Meanwhile, transthoracic echocardiography is the primary modality for diagnosing and assessing MVP, and this technique must include evaluation of the leaflets, annulus, chords, and papillary muscles. Other imaging modalities, such as cardiac MRI and computed tomography (CT), are considered secondary in this regard and are typically used to evaluate the anatomical relationship between the mitral valve annulus and leaflet [26, 27].

**Fig. 2.**
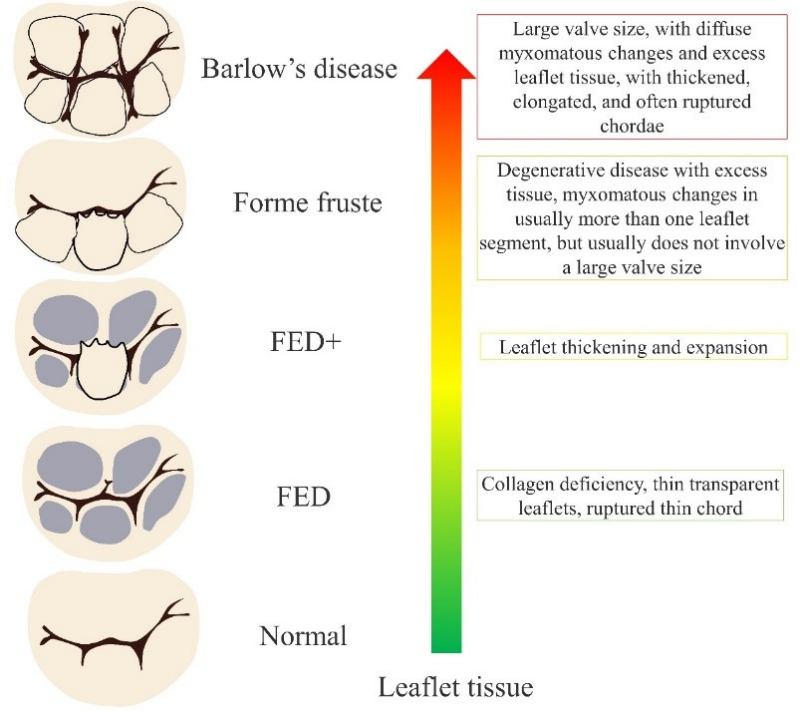
**MVP to Barlow’s disease pathogenesis**. FED, fibroelastic dysplasia.

MVP degeneration encompasses a spectrum of different lesions that may involve a single segment of the valve or a multisegment condition affecting both leaflets. The pathological changes may include chordal rupture, excess tissue, myxomatous changes, tissue expansion, large annular size, etc. The pathogenesis of MVP changes is demonstrated in Fig. 2 [28, 29].

**Fig. 3.**
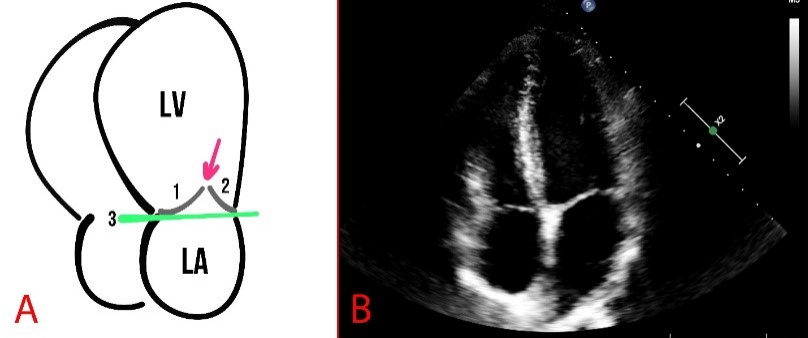
**Schematic presentation (A) and echocardiography (B) of normal findings**. (A) The numbers 1 and 2 show the mitral valve flaps, the line (3) shows the mitral annulus, and the arrow indicates the coaptation zone. LA, left atrium.

The structure and function of the MV leaflets are evaluated polypositionally using different modes, such as M-mode, B-mode, and Color Doppler mode. Normally, the mitral annulus has a curved shape and a saddle-shaped bend in the sagittal plane (Fig. 3).

**Fig. 4.**
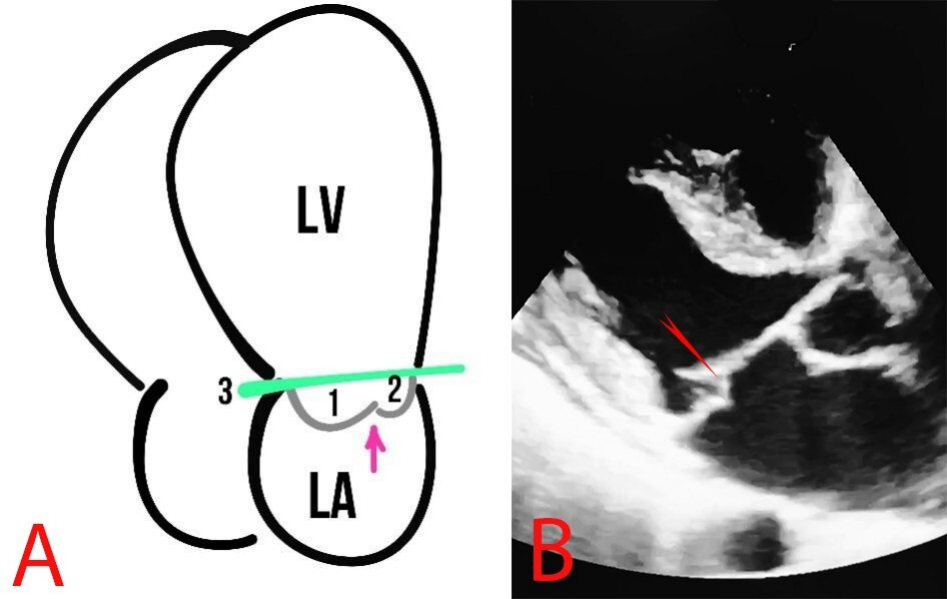
**MVP**. (A) Schematic presentation of MVP. The arrows show the MV, 1 and 2 represent the MV leaflets, the line (3) shows the mitral annulus, and the arrow depicts the coaptation zone. (B) Echocardiography of a patient with MVP, the red arrow indicates MVP.

MVP is defined as the billowing or bulging of MV leaflets more than 2 mm above the mitral annulus in a long-axis view (Fig. 4).

**Fig. 5.**
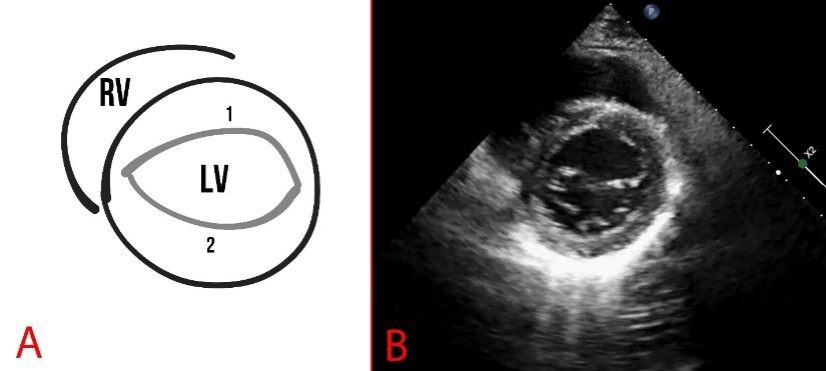
**Schematic representation (A) and echocardiography (B) of parasternal short-axis view at the level of the mitral valve**. A fibrous annulus of the mitral valve is marked, 1 is the anterior leaflet, and 2 is the posterior leaflet of the mitral valve. RV, right ventricle.

**Fig. 6.**
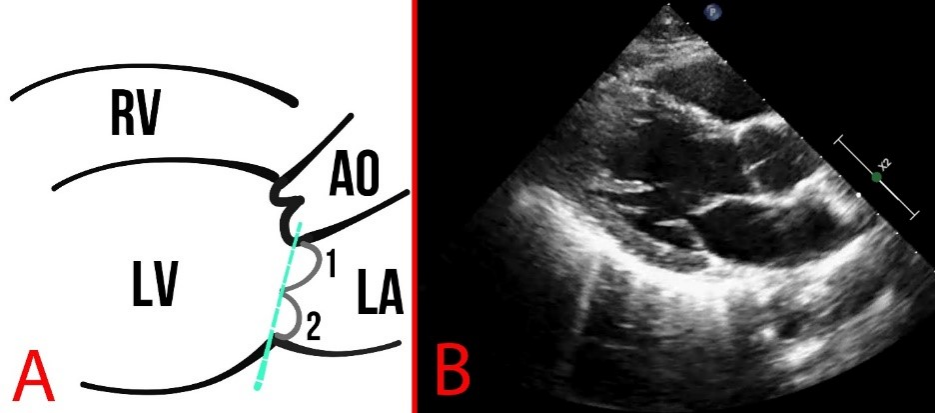
**Schematic representation (A) and echocardiography (B) of normal findings in parasternal long-axis view**. The dotted line indicates the mitral annulus, and numbers 1,2 indicate the leaflets of the MV. AO, aorta.

The anterior leaflet of the mitral valve is larger than the posterior mitral leaflet and more mobile. MVP is assessed during systole (at the moment the valves close) and is considered true when a prolapse is registered in two or more views (Figs. 5,6).

Parasternal short-axis scanning at the level of the mitral valve can reveal pathologies such as splitting of the posterior leaflet into two components, as well as myxomatous degeneration, thickening of the leaflet, convoluted structure, or elongation of the leaflets and chords (Fig. 5).

If MVP is detected, it is recommended to clarify: (1) the view in which the pathology was identified; (2) exact localization of the pathology (anterior, posterior, or both leaflets with indication of a scallop); (3) the distance from the projection of the mitral annulus to the leaflets in millimeters; (4) structural features of the MV (diameter of the mitral annulus and the presence of any deformities, thickening or other lesions of the leaflets).

**Fig. 7.**
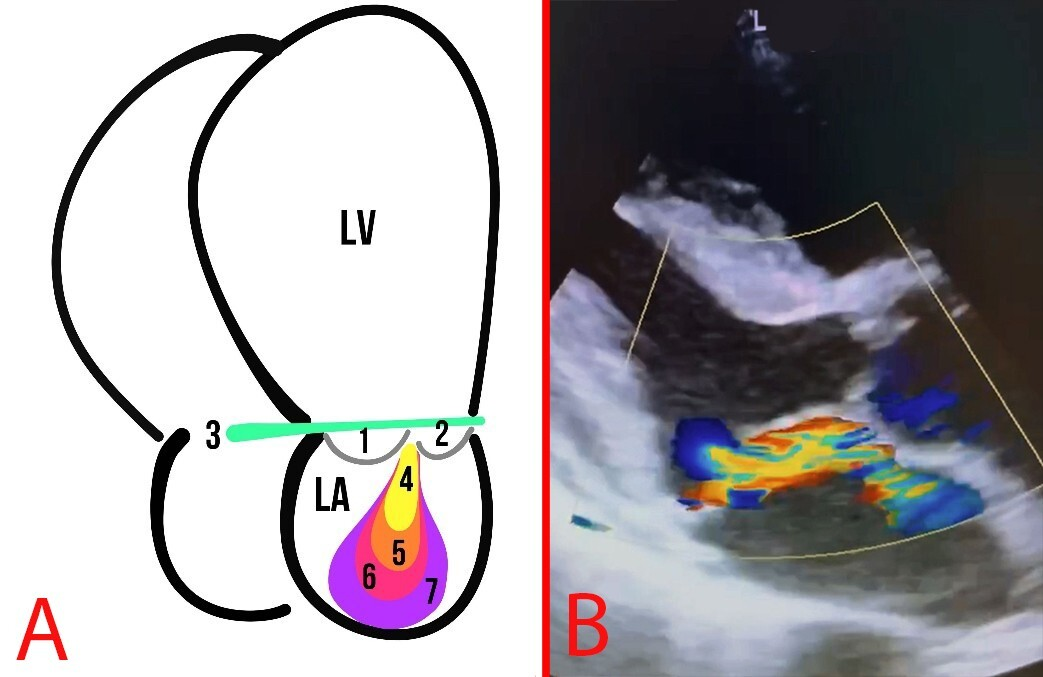
**MR**. (A) Schematic representation of MR grades: The numbers 1 and 2 indicate the MVP leaflets; 3 depicts the mitral annulus line; 4, first degree of MR; 5, second degree of MR; 6, third degree of MR; 7, fourth degree of MR. (B) Echocardiography of a patient with MVP and MR.

Two-dimensional (2D) echocardiography imaging is the modality of choice for evaluating the MR etiology and mechanism. Meanwhile, MR severity is semi-quantitatively assessed by eyeballing the proportion of the left atrium (LA) area occupied by the regurgitant jet on 2D/color Doppler imaging [30]. MVP may result in MR, both acute and chronic. With a prolonged course of the disease, the left atrium changes, becoming dilated due to increased pressure. MR is usually evaluated based on the guidelines of the American Society of Echocardiography (Fig. 7) [31].

A comprehensive assessment of MR in the context of prolapse involves visualizing the mitral valve using a wide range of tools. In M-mode, the patient may have a deflection of one or both valves in the system. In B-mode, it is necessary to assess the dilation of the left chambers of the heart and the mitral annulus, and the function of the mitral valve flaps. In the case of multi-view detection of valve prolapse, the distance from the mitral annular projection is measured in millimeters. Regurgitation assessment: qualitative assessment of the color jet area, vena contracta width (VCW), and the proximal isovelocity surface area (PISA) method. The three methods studied all have known limitations [32]. Current guidelines divide MR into mild, moderate, and severe. Here, severe MR can be diagnosed without extra measurements if four or more criteria are present: fluttering leaflet; vena contracta width >0.7 cm; PISA radius >1.0 cm, central large jet >50% of left atrial area; pulmonary vein systolic flow reversal; normally functioning enlarged left ventricle [31].

It is worth mentioning that the degree of MVP may not be directly proportional to the degree of MR. Indeed, the American Society of Echocardiography (ASE) released a guideline paper on valvular regurgitation in 2017, in which MR can be classified into mild, moderate, and severe categories. The quantity of regurgitation can further subclassify MR into four grades (Table 2) [31].

**Table 2.** **MR regurgitation severity and grades**.

Measure	Grade I	Grade II	Grade III		Grade IV
	Mild	Moderate	Moderate	Severe	Severe
EROA (cm^3^)	<0.20	0.20–0.29	0.30–0.39	0.30–0.39	≥0.4
RVol (mL)	<30	30–44	45–59	45–59	≥60
RF%	<30	30–39	40–49	40–49	≥50

EROA, effective regurgitant orifice area; RVol, regurgitant volume; RF%, regurgitant fraction.

#### 2.2.2 Multimodality Imaging in MVP and MAD

Echocardiography remains the first-line imaging tool due to its cost-effectiveness, wide availability, and ease of repeatability. Moreover, echocardiography can help to assess leaflet morphology, MAD, and MR. Speckle tracking provides insights into abnormal valvular–myocardial mechanics, which is considered the main arrhythmogenic mechanism in MVP [33].

Cardiac MRI provides a detailed characterization of myocardial tissue, enabling the assessment of interstitial fibrosis through late gadolinium enhancement and T1 mapping/extracellular volume fraction [33]. Moreover, cardiac MRI can help define and characterize the composition of the myocardium and identify specific arrhythmic risk factors, such as zones of fibrosis [10, 34]. The arrhythmogenic substrate can include fibrosis of the papillary muscles and the inferobasal left ventricular wall [35, 36]. In a series of 43 cases of SCD in young patients with MVP, papillary muscle fibrosis (88%) or inferobasal fibrosis (93%) was identified. It was also found that the distribution of late gadolinium enhancement on CMR correlated with histopathological findings [10]. During follow-up, patients with a fibrotic pattern were found to tend to develop arrhythmic events [37].

MAD was reported to be a constant component of arrhythmic MVP with LV fibrosis [38]. In a study involving 36 patients with MVP, the MAD was significantly longer (4.8 mm) in those with late gadolinium enhancement (LGE) on MRI compared with those who did not have LGE on MRI. A disjunction length of >8.5 mm was applied to correctly identify 67% of the patients who exhibited no sustained ventricular tachycardia (VT) via Holter monitoring [39].

However, some studies indicate that the prevalence of MAD in patients with MVP was higher, mainly driven by a higher prevalence of pseudo-MAD. Moreover, the extent of pseudo-MAD was greater in patients with MVP than in those without MVP, whereas the extent of true-MAD did not differ significantly [40, 41]. A MAD length of at least 5 mm and coexisting bileaflet MVP showed a higher risk of arrhythmia [40, 41].

Overall, the majority of patients with MVP also appeared to have pseudo-MAD. Meanwhile, true-MAD is a real abnormal attachment of the leaflet to the atrial wall and is visible in both systole and diastole, only in approximately 7% of MVP cases [42]. Cardiac MRI had a higher sensitivity in detecting MAD compared with echocardiography, particularly in cases of MAD of minimal length [43].

## 3. Electrocardiogram (ECG) Changes in MVP

### 3.1 Incidence and Prevalence of Electrocardiogram Changes in MVP

Ventricular ectopic beats (VEBs) are observed in 49–85% of cases using 24-hour Holter monitoring, suggesting that ECG abnormalities are common in patients with MVP [44]. Disease progression, MR, and structural changes, including MAD and bileaflet MVP—both of which increase the risk of VAs—all influence the occurrence of new ECG abnormalities [45, 46].

MVP is observed in approximately 2.59% of people worldwide, although it is more prevalent in syndromic disorders, including Ehlers–Danlos syndrome (8%), Williams–Beuren syndrome (18.36%), and Marfan syndrome (57.16%) [47].

Early repolarization patterns were significantly more common in MVP patients (74%) than in controls (8%), particularly in younger individuals, as reported by Peighambari *et al*. [48]. Early repolarization may be a potential indicator of arrhythmic risk in young patients with MVP, according to this discovery, which is characterized by QRS notching and J-point elevation [48].

Although the precise processes are still being studied, gender variations in MVP also affect ECG abnormalities, with females more likely to display arrhythmogenic MVP phenotypes [46]. However, further research is needed on variances related to ethnicity [47].

### 3.2 Types of ECG Changes Observed in MVP

#### 3.2.1 Atrial Fibrillation

Atrial fibrillation (AF) can lead to atrial dilation and stretching, which, in turn, can promote the induction and sustainability of AF. MVP and MR can promote interstitial fibrosis and inflammation, further facilitating changes in the left atrium. The combination of these factors, including prolapsing leaflets, an expanding annulus, and a dilated atrium, can lead to an MR jet, which can predispose individuals to AF [49]. The vicious cycle between MVP and MR stress on the atrium and continued worsening of the AF burden can lead to further exacerbation of both conditions. The risk of AF increases with age and larger left atrium dimensions in MR patients and is associated with increased mortality [50]. The cornerstones of AF management include rhythm control, rate control, and anticoagulation [51, 52].

#### 3.2.2 Ventricular Arrhythmias

3.2.2.1 Premature Ventricular Complexes (PVCs)

PVCs are often found in MVP, with Holter ECGs showing them in 58% to 89% of patients [53, 54]. The ventricular myocardial tissue is the source of these premature beats, which are distinguished by broad QRS complexes with an irregular shape [55, 56]. PVCs in MVP often show an inferior axis left bundle branch block (LBBB) pattern, indicating an origin from the inferobasal LV or papillary muscles [9, 57, 58]. This pattern, which reflects the shared origin of these ectopic beats within the LV, is also frequently seen in different MVP-related VAs.

VA risk is elevated when the PVC load is large (>10% of total beats) [59]. PVCs induced by exercise and recurrent monomorphic PVCs are considered high-risk indicators that warrant further research [44].

3.2.2.2 Non-Sustained Ventricular Tachycardia (NSVT)

NSVT—defined as three or more consecutive ventricular beats at >100 bpm lasting <30 seconds—is commonly seen in MVP, especially in patients who have frequent PVCs [44, 60, 61]. In line with the results mentioned above, an ECG usually shows the same LBBB pattern with an inferior axis as observed in PVCs [45, 53].

The frequency, length, and exertion-related incidence of isolated NSVT events suggest a propensity for sustained VT or ventricular fibrillation (VF), even if these episodes may not necessarily be clinically important [9, 53].

3.2.2.3 Sustained Ventricular Tachycardia (VT)

Fibrosis and mechanical stress on the mitral valve apparatus are frequently linked to sustained VT in MVP, which is defined as a VA that lasts longer than 30 seconds or necessitates termination because of instability [9, 62, 63]. When MVP-related sustained VT occurs, the ECG frequently exhibits a monomorphic shape, which is again usually an inferior axis LBBB pattern that originates in the papillary muscles or inferobasal LV [10, 45].

TWI in the inferior and lateral leads are additional ECG markers that may be used as early warning signs of arrhythmic risk due to regional repolarization abnormalities [10, 62]. Additionally, myocardial fibrosis and an elevated risk of arrhythmias in MVP have been associated with fragmented QRS (fQRS) [57].

The rate of sustained VT determines the hemodynamic effect [64]. Faster VTs, those >180 bpm, might cause syncope or hypotension, necessitating immediate medical attention, whereas VTs <150 bpm may be tolerated [64]. Cardioversion must be performed immediately to avoid hemodynamic collapse if VT is measured at more than 200 bpm [65].

3.2.2.4 Polymorphic VT and Ventricular Fibrillation

Multiple reentrant circuits form the source of polymorphic VT, which is distinguished by changing QRS shape, fluctuating axis, and uneven cycle duration [9, 19]. Notably, MVP patients with MAD and myocardial fibrosis are more at risk owing to their altered conduction pathways [60]. Meanwhile, the ECG abnormalities that may lead to VF include extended repolarization patterns, short-coupled PVCs, and continuously shifting QRS morphology [57, 66].

Extreme tachycardia (>300 bpm), chaotic fibrillatory waves, and the lack of structured QRS complexes are all signs of VF, a potentially fatal rhythm that can cause cardiac arrest [62, 67]. Fibrosis, as observed by MRI, is a powerful predictor of VF and SCD, especially in the posterior papillary muscle [53].

#### 3.2.3 Repolarization Abnormalities

T-Wave Inversion in Inferior and Lateral Leads

TWI in the inferior (II, III, aVF) and lateral (I, aVL, V5, V6) leads is used to characterize arrhythmogenic MVP, which indicates aberrant ventricular repolarization and elevated arrhythmic risk [44, 60]. Although the exact processes behind TWI in MVP are not entirely known, these progressions may include localized fibrosis that affects repolarization, modest structural abnormalities, or regional myocardial strain resulting from abnormal leaflet motion [9]. Certain genetic conditions, such as Noonan syndrome, also present with characteristic ECG abnormalities that might influence repolarization patterns [68, 69].

To aid in risk classification, TWI is often associated with early repolarization patterns, QTc prolongation, and fQRS [57]. Although further research is needed to quantify this risk precisely, studies have demonstrated that TWI in the inferior leads, especially when associated with MAD, is a powerful predictor of adverse outcomes in patients with MVP [70].

In a study of MVP patients who died of SCD, inferior lead TWI was noted in 83% of the available ECGs [10]. Therefore, observing TWI is essential to identify individuals with MVP who are most at risk for potentially fatal arrhythmias, particularly when combined with other ECG abnormalities.

### 3.3 Risk Stratification and Clinical Consequences

Since everyone has a different risk of adverse cardiac events, including SCD, risk stratification is essential in the treatment of MVP [10]. Thus, identifying MVP patients who are most at risk for potentially fatal arrhythmias and other consequences represents the aim of risk stratification, which enables the implementation of suitable therapies [71].

#### 3.3.1 Cardiac Dysfunction

Left ventricular dysfunction (LVD) may be exacerbated by MVP-related arrhythmias, especially frequent PVCs [53]. Moreover, PVCs can even promote PVC-induced cardiomyopathy [72]. Furthermore, with a sensitivity of 79% and specificity of 78% for the occurrence of cardiomyopathy, a PVC load of more than 24% has been identified as a significant predictor of this syndrome [72]. Crucially, PVC-induced cardiomyopathy is frequently curable; ventricular dysfunction can be significantly improved or even resolved with early detection and treatment of the elevated PVC load [12, 73]. The numerous ectopic beats are thought to be caused by structural and electrical remodeling of the heart [74]. Although PVCs are the most frequent cause, further study is required to determine whether other arrhythmias, such as NSVT, may contribute to LVD [9, 13, 53]. Thus, evaluating cardiac function, particularly when assessing for PVC-induced cardiomyopathy, is a crucial part of risk classification for patients with MVP.

#### 3.3.2 Acute Coronary Syndrome (ACS)

The diagnosis of MVP can be difficult because ECG abnormalities such as TWI and ST-segment depression might resemble ischemia patterns found in ACS [75, 76]. These repolarization anomalies should be thoroughly evaluated to exclude concurrent ischemia, even though these anomalies are frequently associated with myocardial strain and fibrosis rather than coronary artery disease [57]. Meanwhile, it is crucial to distinguish between ECG alterations caused by ACS and MVP [76]. Particularly in older individuals with conventional risk factors for atherosclerosis, the possibility of concomitant CAD should be considered [77]. Additional tests, including stress tests or coronary angiography, can be required if there is a clinical suspicion of ACS [75]. Further investigation is needed to establish a definitive connection, despite some researchers theorizing that arrhythmic MVP may indirectly increase the risk of ACS through mechanisms such as increased mechanical stress or endothelial dysfunction [78]. Thus, understanding the possibility that MVP-related ECG alterations might resemble ACS is essential for an accurate diagnosis and suitable treatment.

#### 3.3.3 Ventricular Fibrillation and Sudden Cardiac Death

Although relatively uncommon, VF and SCD are among the most dreaded side effects of MVP. Certain ECG findings (polymorphic VT, short-coupled PVCs, prolonged QTc interval, TWI in inferior/lateral leads), clinical history (previous syncope, family history of SCD), imaging findings (MAD, myocardial fibrosis), and possibly specific genetic syndromes are among the risk factors that have been identified [12]. A prior systematic review by Han *et al*. [19] found that the incidence of SCD in MVP was around 217 occurrences per 100,000 person-years; however, the risk varied greatly among subgroups; for instance, patients with MAD and a history of syncope were found to be significantly more vulnerable [23]. Ultimately, accurate risk classification that takes these aspects into account is crucial to inform management choices, such as more frequent monitoring, lifestyle changes, medication, or the insertion of an implanted cardioverter-defibrillator (ICD) in high-risk patients.

## 4. Pharmacological Management of Arrhythmias in MVP

### 4.1 Introduction

Pharmacological therapy is essential to treat the symptoms and reduce the dangers related to arrhythmias in MVP [53, 79]. A small percentage of people with MVP exhibit various arrhythmic symptoms, ranging from palpitations and lightheadedness to syncope and, in rare instances, SCD; meanwhile, most remain asymptomatic [12, 80]. Pharmacological therapy for symptomatic MVP-related arrhythmias aims to: (1) reduce the frequency and severity of arrhythmic episodes, preventing progression to more complex arrhythmias; (2) alleviate distressing symptoms, such as palpitations, chest pain, and lightheadedness; (3) reduce the risk of serious complications, such as SCD and thromboembolic events [60, 62, 81]. Pharmacological therapies focus on the structural abnormalities, such as MAD, repolarization abnormalities, and autonomic dysfunction, which are underlying electrophysiological processes that cause cardiac arrhythmias [60]. This focused strategy in patients with symptomatic arrhythmic MVP seeks to enhance quality of life, restore heart rhythm stability, and eventually lower morbidity and death [82]. This section will cover the various drug classes used to treat arrhythmias associated with MVP, including their mechanisms of action, unique functions in this disease, and key usage considerations.

### 4.2 Antiarrhythmic Drugs in the Management of AMVP

The pharmacological treatment of arrhythmias linked to MVP heavily relies on antiarrhythmic medications. The Vaughan-Williams categorization method is most frequently used to classify these drugs based on their main electrophysiological effects [83]. Although this classification provides a helpful foundation, it is essential to recognize that many medications have multiple mechanisms of action [84]. Understanding these processes is crucial in the context of AMVP, as it enables the selection of the most effective treatment for each patient.

#### 4.2.1 Class I Antiarrhythmic Drugs (Sodium Channel Blockers)

Sodium channel blockers, a family of antiarrhythmic medications, inhibit fast sodium channels in cardiac myocytes. Mostly affecting tissues with high rates of depolarization, this effect decreases conduction velocity and delays phase 0 depolarization [85]. Overall, sodium channel blockers are used to treat specific arrhythmias in the setting of AMVP when alternative treatments, including beta-blockers, are unsuccessful or inappropriate [79].

4.2.1.1 Class IC Antiarrhythmics

VAs are treated with sodium channel blockers, such as flecainide and propafenone, which belong to the class IC medication group [85, 86, 87]. With no impact on the length of action potentials, these compounds demonstrate strong sodium channel blockage capabilities [85]. These medications are also helpful for AMVP patients who have palpitations, PVCs, or non-sustained VT because these compounds can effectively suppress both the supraventricular and VA [12, 80].

However, patients with structural cardiac disease, such as severe MR or LV dysfunction, which can occasionally be present in AMVP, are particularly at risk for proarrhythmias while taking Class IC medications [86, 87, 88]. Subsequently, these medications should be taken with caution for AMVP, and patients should be regularly watched for the emergence of new or worsening arrhythmias.

4.2.1.2 Class IA and IB Antiarrhythmics

Class IA medications, such as quinidine and procainamide, increase the risk of torsades de pointes, a potentially fatal arrhythmia, by prolonging the length of the action potential and the QT interval [85, 89, 90]. Class IA medications are rarely used to treat AMVP because of this risk and the availability of safer alternatives. Class IB medications, including lidocaine and mexiletine, are often less successful in treating persistent arrhythmias because these medications predominantly attack ischemic or depolarized tissue [85, 91, 92]. Moreover, these compounds play a minor role in AMVP and are primarily reserved for acute VA under specific conditions.

#### 4.2.2 Class II Antiarrhythmic Drugs (Beta-Blockers)

Beta-blockers are essential to manage certain elements of AMVP, as these compounds inhibit beta-adrenergic receptors [79]. These medications decrease sympathetic activity, which can be elevated in certain MVP patients and lead to arrhythmias and related symptoms [80]. Beta-blockers are especially helpful in treating symptoms of elevated adrenergic tone in AMVP, including anxiety, palpitations, and chest discomfort [82]. PVCs and other supraventricular arrhythmias can also be less common following treatment with beta-blockers [12].

4.2.2.1 Specific Beta-Blockers in AMVP

Notably, cardioselective beta-blockers, such as metoprolol and bisoprolol, are typically recommended in AMVP because these medications are less likely to cause bronchospasm and other beta-2-mediated adverse effects [93, 94, 95]. Indeed, these substances can efficiently lower heart rate and contractility without significantly impacting the airways by targeting beta-1 receptors in the heart [93, 94, 95]. In certain situations where anxiety or other beta-2-mediated symptoms are significant, non-selective beta-blockers, such as propranolol, may be considered; however, patients with asthma or other respiratory disorders should use these medications with caution [96, 97].

4.2.2.2 Considerations for Beta-Blocker Use in AMVP

Although beta-blockers can help control symptoms and lower the incidence of some arrhythmias in AMVP, these compounds are not always successful in completely stopping all arrhythmias, particularly when notable structural abnormalities are present, such as MAD [53, 80]. Thus, in these situations, additional antiarrhythmic drugs or other procedures, such as catheter ablation, may be required [53, 80].

#### 4.2.3 Class III Antiarrhythmic Drugs (Potassium Channel Blockers)

Potassium channel blockers, also known as Class III antiarrhythmics, inhibit potassium channels, thereby increasing the duration of action potentials and the effective refractory period in cardiac myocytes [85]. Certain arrhythmias linked to MVP, especially those involving reentry processes, may benefit from this treatment [79].

4.2.3.1 Amiodarone in AMVP

Amiodarone is the most widely utilized potassium channel blocker for arrhythmic MVP treatment [12]. Amiodarone has a wide range of antiarrhythmic actions, impacting beta-adrenergic receptors, potassium channels, sodium channels, and calcium channels [85, 98]. Meanwhile, amiodarone has a complex mechanism of action, which can effectively suppress VT, AF, and PVCs, among other atrial and VAs that may arise in AMVP [80]. However, the possibility of severe extracardiac toxicity, which can impact the thyroid, lungs, liver, and eyes, limits the long-term use of amiodarone [98, 99]. Therefore, amiodarone is usually saved for AMVP patients with arrhythmias that are symptomatic and have not responded to other antiarrhythmic medications, or when other treatments are not appropriate [12, 100].

4.2.3.2 Other Class III Antiarrhythmics

Compared to amiodarone, other Class III antiarrhythmics, such as sotalol, dofetilide, and ibutilide, are used less frequently in AMVP [12]. Indeed, compared to amiodarone, these medications have a greater risk of torsades de pointes and QT interval lengthening since they mainly impact potassium channels [85, 101, 102, 103]. Additionally, sotalol possesses beta-blocking properties, which some AMVP patients may find beneficial [104]. However, the proarrhythmic risk of these medicines usually restricts their use in AMVP, especially in patients with structural heart disease or other QT prolongation risk factors [100, 101, 102, 103].

4.2.3.3 Considerations for Potassium Channel Blocker Use in AMVP

Potassium channel blockers should be used cautiously in AMVP due to the potential for severe adverse effects, particularly with prolonged treatment [98, 105]. Patients should be cautiously watched for the onset of extracardiac toxicity, torsades de pointes, and QT prolongation [98, 100, 105]. Meanwhile, routine monitoring of liver function, lung function, and thyroid function is advised for patients on long-term amiodarone treatment [98, 99].

#### 4.2.4 Class IV Antiarrhythmic Drugs (Calcium Channel Blockers)

Heart rate, contractility, and vascular tone are all reduced by calcium channel blockers (CCBs), which block L-type calcium channels in cardiac myocytes and vascular smooth muscle [85]. Overall, CCBs have a specific but limited role in the treatment of AMVP, especially non-dihydropyridine (non-DHP) CCBs [79].

4.2.4.1 Non-Dihydropyridine CCBs in AMVP

Certain supraventricular tachycardias (SVTs) that may develop in AMVP, such as AF with a fast ventricular response, can be controlled by non-DHP CCBs, such as verapamil and diltiazem, which mainly influence cardiac conduction [80, 106, 107]. These medications can help control heart rate by slowing conduction through the AV node [106, 107]. However, these medications are typically less successful than beta-blockers when it comes to suppressing the underlying arrhythmias linked to AMVP, including PVCs or non-sustained ventricular tachycardia [12, 106].

4.2.4.2 Dihydropyridine CCBs in AMVP

Amlodipine and nifedipine are examples of dihydropyridine CCBs that mainly function as vasodilators and have limited impact on cardiac conduction [85]. Thus, dihydropyridine CCBs are rarely employed in treating arrhythmias linked to AMVP [12, 79]. Moreover, dihydropyridine CCBs do not directly address the arrhythmic processes or symptoms associated with MVP, but may be utilized to manage concomitant hypertension [12, 79].

4.2.4.3 Considerations for CCB Use in AMVP

Non-DHP CCBs are often not the first-line treatment for managing arrhythmias, but these medications may be useful for rate control in certain SVTs associated with AMVP [12, 106, 107]. Meanwhile, beta-blockers are frequently recommended for symptom management and arrhythmia suppression in AMVP [12]. Additionally, because CCBs might further decrease contractility, these medications should be administered cautiously in patients with LV failure [108, 109].

Table 3 (Ref. [84, 87, 89, 90, 91, 92, 93, 94, 95, 97, 98, 100, 101, 102, 103, 105, 106, 107, 110, 111, 112]) summarizes the key pharmacological agents used in managing arrhythmias associated with MVP, including their mechanisms of action, target channels, indications, contraindications, common side effects, and special considerations for their use (Table 3).

**Table 3.** **Antiarrhythmic drugs in AMVP**.

Drug/class	Mechanism of action	Channels affected	Indications	Contraindications	Key side effects	Special considerations	References
Class IC: Flecainide	Blocks sodium (Na^+^) channels, thereby slowing conduction velocity in the atria, ventricles, and Purkinje fibers.	Na^+^	Supraventricular arrhythmias, including AF, SVT, and other rhythm disorders.	Structural heart disease (e.g., CAD, cardiomyopathy), heart failure (especially with reduced ejection fraction), Brugada syndrome, and recent myocardial infarction.	Proarrhythmia (VT), dizziness, blurred vision, and dyspnea.	Avoid administration in structural heart disease. Monitor ECG for QRS widening.	[100, 105, 110, 111]
Class IC: Propafenone	Blocks sodium (Na^+^) channels, thereby slowing conduction velocity. Exhibits weak beta-blocking activity.	Na^+^	Paroxysmal atrial fibrillation, SVT, and other supraventricular arrhythmias.	Structural heart disease, heart failure (especially with reduced ejection fraction), severe bradycardia, and significant conduction disturbances.	Metallic taste, constipation, proarrhythmia, and dizziness.	CYP2D6 inhibitor; adjust doses in hepatic impairment. Monitor ECG for QRS widening.	[87, 100, 105]
Class IA: Quinidine	Blocks sodium (Na^+^) channels, thereby slowing conduction, and blocks potassium (K^+^) channels, thereby prolonging repolarization.	Na^+^ and K^+^	AF, VA, and other rhythm disorders.	Long QT syndrome, heart block, myasthenia gravis, history of torsades de pointes.	Cinchonism (tinnitus, headache, visual disturbances), torsades de pointes, and gastrointestinal disturbances.	Monitor QT interval; risk of torsades de pointes. Interacts with digoxin and warfarin.	[89, 100, 105, 110]
Class IA: Procainamide	Blocks sodium (Na^+^) channels, thereby slowing conduction.	Na^+^	VA, WPW syndrome, and other arrhythmias.	SLE, heart block, torsades de pointes, and hypersensitivity to procainamide.	Lupus-like syndrome, hypotension, proarrhythmia, gastrointestinal disturbance.	Monitor for lupus-like symptoms. Avoid long-term use.	[90, 100, 105, 110]
Class IB: Lidocaine	Blocks sodium (Na^+^) channels, preferentially in ischemic tissue, thereby shortening the action potential duration.	Na^+^	Acute VA (e.g., ventricular tachycardia, ventricular fibrillation), and other acute cardiac rhythm disturbances.	Severe bradycardia, heart block, and hypersensitivity to lidocaine.	Central nervous system effects (dizziness, seizures), hypotension, and arrhythmias.	Use with caution in liver dysfunction; monitor for CNS toxicity.	[91, 100, 105, 110]
Class IB: Mexiletine	Blocks sodium (Na^+^) channels, similar to lidocaine.	Na^+^	VA, chronic pain (off-label), and other conditions.	Cardiogenic shock, severe heart failure, and known hypersensitivity to mexiletine.	Nausea, tremor, dizziness, and proarrhythmia.	Adjust dose in renal/hepatic impairment; monitor ECG.	[92, 100, 105, 110]
Class II: Metoprolol	Selective beta-1 (β1) adrenergic receptor blockade, thereby decreasing heart rate and contractility.	Beta-1 adrenergic receptors	Rate control in AF, SVT, VA, and other conditions where beta-blockade is indicated.	Severe bradycardia, heart block, decompensated heart failure, acute myocardial infarction, and active bronchospasm (relative contraindication).	Bradycardia, fatigue, hypotension, and bronchospasm.	Use with caution in asthma/COPD. Monitor heart rate and BP.	[84, 93, 94, 112]
Class II: Bisoprolol	Selective beta-1 (β1) adrenergic receptor blockade, thereby decreasing heart rate and contractility.	Beta-1 adrenergic receptors	Rate control in AF, SVT, VA, and other conditions where beta-blockade is indicated.	Severe bradycardia, heart block, cardiogenic shock, decompensated heart failure.	Fatigue, dizziness, bradycardia, and hypotension.	Adjust dose in renal/hepatic impairment. Monitor for bradycardia.	[84, 93, 95, 112]
Class II: Propranolol	Non-selective beta (β1 and β2) adrenergic receptor blockade, thereby decreasing heart rate and contractility.	Beta-1 and beta-2 adrenergic receptors	Rate control in AF, SVT, VA, and other conditions where beta-blockade is indicated.	Asthma, severe bradycardia, heart block, decompensated heart failure, and acute myocardial infarction.	Bradycardia, fatigue, depression, bronchospasm, and hypoglycemia.	Avoid in asthma. Monitor for CNS side effects.	[84, 93, 97]
Class III: Amiodarone	Complex mechanism: Blocks potassium (K^+^), sodium (Na^+^), and calcium (Ca^2+^) channels; exhibits non-competitive beta-adrenergic blockade and other effects. Prolongs action potential duration and refractoriness.	K^+^, Na^+^, Ca^2+^	AF, VT, VF, and other refractory arrhythmias.	Severe bradycardia, sinoatrial block, thyroid dysfunction (relative contraindication), and known hypersensitivity to amiodarone.	Pulmonary toxicity (pneumonitis, fibrosis), thyroid dysfunction (hypothyroidism, hyperthyroidism), hepatotoxicity, QT prolongation, and bradycardia.	Monitor LFTs, TFTs, and CXR. Long half-life (weeks to months). Risk of QT prolongation and torsades de pointes.	[84, 98, 100, 105]
Class III: Sotalol	Blocks potassium (K^+^) channels, prolonging repolarization, and exhibits non-selective beta (β1 and β2) adrenergic blockade.	K^+^	AF, VT, VF, and other arrhythmias.	Long QT syndrome, severe bradycardia, decompensated heart failure, renal impairment (use with caution).	Torsades de pointes, bradycardia, fatigue, and QT prolongation.	Monitor QT interval; risk of torsades de pointes. Adjust the dose in renal impairment.	[84, 100, 101, 110]
Class III: Dofetilide	Selective blockade of potassium (K^+^) channels, prolonging repolarization.	K^+^	AF, atrial flutter.	Long QT syndrome, severe renal impairment.	Torsades de pointes, headache, dizziness, and QT prolongation.	Requires dose adjustment based on CrCl. Monitor QT interval; risk of torsades de pointes.	[84, 102, 110]
Class III: Ibutilide	Appears to prolong action potential duration by blocking potassium (K^+^) channels and/or activating slow sodium (Na^+^) channels.	K^+^	Acute conversion of AF or atrial flutter to normal sinus rhythm.	Long QT syndrome, severe heart failure.	Torsades de pointes, hypotension, nausea, and QT prolongation.	Administer in a monitored setting. Monitor QT interval; risk of torsades de pointes.	[84, 100, 103]
Class IV: Verapamil	Blocks L-type calcium (Ca^2+^) channels, thereby reducing myocardial contractility and slowing AV node conduction.	Ca^2+^	Rate control in AF, SVT, and other conditions.	Severe heart failure, heart block, hypotension, WPW syndrome with accessory pathway conduction.	Hypotension, bradycardia, constipation, and peripheral edema.	Avoid in heart failure. Monitor BP and heart rate.	[84, 100, 105, 106]
Class IV: Diltiazem	Blocks L-type calcium (Ca^2+^) channels, similar to verapamil, but with relatively less effect on contractility.	Ca^2+^	Rate control in AF, SVT, and other conditions.	Severe heart failure, heart block, hypotension, atrial fibrillation/flutter with rapid ventricular response, and an accessory pathway.	Hypotension, bradycardia, peripheral edema, and headache.	Use with caution in liver dysfunction. Monitor BP and heart rate.	[84, 100, 107]

Footnotes: AF, atrial fibrillation; VA, ventricular arrhythmia; SVT, supraventricular tachycardia; CAD, coronary artery disease; ECG, electrocardiogram; VT, ventricular tachycardia; CYP2D6, cytochrome P450 2D6; WPW, Wolff–Parkinson–White; SLE, systemic lupus erythematosus; CNS, central nervous system; COPD, chronic obstructive pulmonary disease; BP, blood pressure; LFTs, liver function tests; TFTs, thyroid function tests; CXR, chest X-ray; CrCl, creatinine clearance.

### 4.3 Adjunctive Pharmacological Therapies in the Management of AMVP

Numerous other pharmacological treatments, in addition to antiarrhythmic medications, represent crucial supplementary measures in the overall treatment of AMVP. These treatments target particular elements of the illness, including MR, LV dysfunction, thromboembolic risk, and related symptoms.

#### 4.3.1 Anticoagulant Therapy in AMVP

Patients with AMVP who also possess AF or other thromboembolism risk factors, such as a history of stroke or transient ischemic attack, should take anticoagulants [12, 79]. Furthermore, these individuals should have their stroke risk evaluated using the CHA_2_DS_2_-VASc score [51]. Except for some circumstances, such as individuals with severe mitral stenosis or artificial heart valves, direct oral anticoagulants (DOACs) are often recommended over warfarin because of their simplicity of administration and decreased risk of significant bleeding [51, 113, 114]. Anticoagulation is crucial for avoiding stroke and other thromboembolic consequences in high-risk AMVP patients, but it does not directly treat the arrhythmias linked to MVP [12, 79].

#### 4.3.2 ACE Inhibitors and ARBs in AMVP

The arrhythmias of AMVP are not usually treated immediately with angiotensin-converting enzyme inhibitors (ACE inhibitors) or angiotensin receptor blockers (ARBs). The main responsibility of these medications is to treat patients with substantial MR or LV dysfunction in addition to AMVP [79, 115, 116]. In these situations, ARBs and ACE inhibitors help lower afterload, halt ventricular remodeling, and delay the progression of heart failure [117]. In AMVP patients with concurrent MR or heart failure, these drugs can indirectly enhance symptoms and quality of life by enhancing cardiac function [117].

#### 4.3.3 Diuretics in AMVP

In AMVP, diuretics are used to treat symptoms of volume overload, which can arise when there is a significant amount of MR [12, 79, 117]. Diuretics ease symptoms, including lung congestion and dyspnea, and lower preload [118]. Nonetheless, diuretics cannot be used to treat the arrhythmias or the underlying valvular condition; the primary purpose of diuretics is to alleviate symptoms, and these medications should be an integral part of a comprehensive management plan that addresses the root cause of the volume overload [118].

### 4.4 Emerging Therapies and Research in AMVP

The goals of the ongoing research on AMVP include improved risk assessment, a deeper understanding of the underlying pathophysiology, and the development of tailored treatments. Although recognized antiarrhythmic medications and adjuvant therapies are the mainstay of current pharmacological care, newer techniques offer more individualized and efficient treatment plans.

#### Pharmacological Research Directions in AMVP

Several promising areas of pharmacological research are being explored for AMVP:

Targeted Antiarrhythmic Therapies: The goal of current research is to develop medications that specifically target the pathophysiological processes linked to arrhythmias associated with AMVP. This includes research on medications that can alter ion channels known to play a role in arrhythmogenesis in the setting of MVP, as well as treatments aimed at reducing myocardial fibrosis, which is believed to be a contributing factor to arrhythmogenicity in some patients with MVP [10, 57, 60].

Genetic and Biomarker-Based Risk Stratification: Research is being conducted to identify blood biomarkers and genetic markers that can predict the risk of sudden cardiac death and other severe arrhythmic events in individuals with AMVP [57, 119]. Thus, by identifying which patients are most likely to benefit from early therapies, such as ICDs or more aggressive pharmaceutical treatments, these studies aim to tailor treatment plans.

Improved Imaging and Electrophysiological Techniques: To further describe the structural and electrophysiological anomalies linked to AMVP, advanced imaging modalities, including cardiac MRI and electroanatomic mapping, are being employed [60]. These methods may direct the development of more targeted pharmaceutical treatments or aid in identifying specific targets for catheter ablation.

## 5. Surgical Repair of MVP

Several case reports have demonstrated that mitral valve surgery can reduce the burden of VA; however, this reduction varies from case to case [120, 121, 122, 123]. It appears that marked anatomical, genetic, and pathophysiological changes in the heart can decrease the likelihood of achieving stable rhythm control after surgery [119, 123].

MV replacement was a high-risk procedure with a mortality of 20–30% in the 1960s. Introduction of new techniques such as ring annuloplasty, leaflet reconstruction, and chordal shortening/transfer leads to the establishment of MV repair as the procedure of choice in symptomatic mitral disease [124]. However, debates remain about whether mitral valve surgery is beneficial in patients with symptomatic MVP.

A retrospective analysis of 4477 patients from the Mayo Clinic who underwent MV surgery demonstrated that eight patients had an ICD in place both pre- and post-surgery. The study of this case series showed a reduction in VF, VT, and ICD shocks after MV surgery [125]. Several successful reports led to the assumption that surgical correction may improve the lives of patients with arrhythmic MVP, leading to a number of new studies involving a larger number of patients.

Ascione and coworkers [126] demonstrated in their study of 29 arrhythmogenic MVP that 45% remained arrhythmogenic after surgery, while 55% became non-arrhythmogenic. Interestingly, Ascione *et al*. [126] also had a non-arrhythmogenic MVP group, in which 18.6% developed arrhythmias after surgery. Patients who experienced arrhythmia reduction had a higher prevalence of MAD compared with those who remained arrhythmogenic (63.6% vs. 11.1%; *p* = 0.028) [126].

A similar study of 32 patients undergoing MV surgery for MR secondary to bileaflet MVP between 1993 and 2012 at the Mayo Clinic demonstrated that VA burden decreased by at least 10% after the surgery in 53.1% of cases. Patients who had a reduction in VA burden were younger (<60 years), suggesting that early surgical intervention may modify the underlying electrophysiologic substrate [127].

Cavigli and coworkers [128] evaluated 23 patients with severe MR due to MVP, 26% of whom had complex VA, and 56.5% of patients had late gadolinium enhancement of the papillary muscles and/or of the basal inferolateral wall. After the surgery for MVP, there was no significant reduction in VA [128].

A more detailed presentation of the results from the available studies is presented in Table 4 (Ref. [14, 16, 125, 126, 127, 128, 129]).

**Table 4.** **Studies assessing cardiac surgery in patients with MVP**.

Author, year	Method	Results
Reece and coworkers, 1985 [129]	A total of 37 symptomatic patients with mitral systolic click	A total of 62% of patients with MVP alone and 91% with associated regurgitation exhibited improvements of at least one New York Heart Association Functional class, and 60% of patients obtained relief of one or more symptoms.
Grigioni and coworkers, 1999 [14]	The occurrence of SCD was analyzed in 348 patients with MVP and MR diagnosed echocardiographically	Surgical correction of MR (n = 186) was independently associated with a reduced incidence of SCD (adjusted hazard ratio [95% confidence interval] 0.29 [0.11 to 0.72]; *p* = 0.007).
Enriquez-Sarano and coworkers, 2005 [16]	A total of 456 patients with asymptomatic organic MR	Cardiac surgery was performed in 232 patients and was independently associated with improved survival (adjusted risk ratio, 0.28; 95% confidence interval, 0.14 to 0.55; *p * < 0.01).
Vaidya and coworkers, 2016 [125]	A retrospective analysis of 4477 patients from the Mayo Clinic who underwent MV surgery demonstrated that eight patients had ICD	Among these patients, there was a reduction in VF (0.6 vs. 0.14 events per-person-year pre- and post-surgery, respectively), VT (0.4 vs. 0.05 events per-person-year pre- and post-surgery, respectively), and ICD shocks (0.95 versus 0.19 events per-person-year pre- and post-surgery) following mitral valve surgery.
Naksuk and coworkers, 2016 [127]	A total of 32 patients undergoing MV surgery for MR secondary to biMVP	VE burden was unchanged after the surgery (*p* = 0.34). However, in 17 patients (53.1%), VE burden decreased by at least 10% after the surgery. These patients were younger (59 ± 15 vs. 68 ± 7 years; *p* = 0.04).
Ascione and coworkers, 2023 [126]	A total of 88 patients with Barlow’s disease. At baseline, 29 patients (33%) were arrhythmogenic (AR), while 59 (67%) were not (non-arrhythmogenic (NAR))	Among AR patients, nine (45%) remained AR after mitral surgery, while 11 (55%) became NAR. Considering NAR subjects at baseline, after mitral valve repair, eight (18.6%) evolved into AR, while 35 (81.4%) remained NAR.
Cavigli and coworkers, 2024 [128]	The study included 23 patients. Six (26%) patients had pre-operative episodes of complex VA (sustained and non-sustained ventricular tachycardia)	After the surgery, no significant reduction in VA was observed, neither in terms of arrhythmic burden nor complexity.

SCD, sudden cardiac death; ICD, implanted cardioverter-defibrillator.

A prior study also presented evidence that the burden of AF can be improved by MV surgery combined with the Maze IV procedure [130]. The study included 64 patients with degenerative mitral insufficiency complicated by AF, 56 (86%) of whom had sinus rhythm 14 months after surgery.

Heart valve surgery is currently the second most common type of cardiac surgery, accounting for 20% to 35% of all cardiac surgical procedures [131].

The current data indicate that the contemporary mortality risk of MV surgery is less than 1% for the majority of patients. The mortality risk is less than 0.5% in patients younger than 65 years, and 97% of the total evaluated population across age groups have a risk of less than 3%. Only a few patients aged 75 or older had a mortality risk of more than 3% [132]. This demonstrated that MV repair is a standardized procedure with a relatively low risk of postoperative mortality. Major complications are also rare, and thromboembolism is seen in approximately 1%, bleeding in <0.5% and endocarditis in <0.5% [133].

Nevertheless, MV surgery represents a major surgery, especially in older patients with multiple comorbidities. It seems that MV surgery, through suppressing the progression of MVP, may have a role in preventing SCD by reducing VA burden. However, the data are inconsistent and mainly derived from case series. Based on the available data and evaluation of clinical phenotypes of MVP, the surgical approach to MVP is currently not proposed in patients with high-risk VAs without severe MR [12, 125, 127, 134]. It should also be noted that non-AMVP can develop arrhythmias after surgery in 18.6% of cases [126].

Another major challenge for every surgical field is the variation in surgical techniques. For instance, leaflet coaptation moving toward the apex and below the annular plane may abolish the prolapse-triggered stretch on the papillary muscles. However, this method is not always applicable, and proper comparison between different techniques is usually not performed [126].

### 5.1 Percutaneous Repair of MV

Notably, cardiac surgery can be associated with increased mortality in a subgroup of patients. Over the years, several transcatheter therapies have been developed to overcome the increased number of subjects with symptomatic severe MR and high surgical risk, which can vary from one study to another, and may be related to the experience of the operator and MV complexity [135].

Several trials have shown that the percutaneous edge-to-edge procedure, the M-TEER, is safe (Table 5, Ref. [136, 137, 138, 139]). Although percutaneous repair was less effective at reducing MR than conventional surgery, the procedure was associated with superior safety and similar improvements in clinical outcomes and resulted in a lower rate of hospitalization for heart failure and lower all-cause mortality within 24 months of follow-up than medical therapy alone [136, 137]. The COAPT study analyzed patients with secondary mitral valvulopathy and heart failure, not with primary valve pathology [137]. However, the results of the study can be extrapolated to some degree to primary valve pathology.

**Table 5.** **Percutaneous repair of MV in case of MR**.

Author, year	Method	Results
Feldman and coworkers, 2011 [136]	Randomly assigned 279 patients with moderately severe or severe MR	Percutaneous repair was less effective at reducing MR than conventional surgery. However, the procedure was associated with superior safety and similar improvements in clinical outcomes.
Grasso and coworkers, 2013 [138]	A total of 117 patients were included in the study	Freedom from death, surgery for mitral valve dysfunction, or grade ≥3+ MR was 96.4% and 75.8% at 30 days and 1 year, respectively.
Maisano and coworkers, 2013 [139]	A total of 567 patients with significant MR underwent M-TEER therapy at 14 European sites	The M-TEER implant rate was 99.6%. A total of 19 patients (3.4%) died within 30 days after the M-TEER procedure. The Kaplan–Meier survival outcome at 1 year was 81.8%.
Stone and coworkers, 2018 [137]	A total of 614 patients were enrolled in the trial, with 302 assigned to the device group and 312 to the control group	Transcatheter mitral valve repair resulted in a lower rate of hospitalization for heart failure and lower all-cause mortality within 24 months of follow-up than medical therapy alone.

### 5.2 Catheter Ablation in Arrhythmias and Mitral Prolapse

Evaluation of the evidence suggests that concomitant ablation for AF during mitral valve surgery is both safe and efficacious. The authors included 36 studies in their meta-analysis, involving 8340 patients. The pooled effectiveness of the results was 76.9%. It is worth noting that the results were associated with significant heterogeneity, reflecting variations in institutional protocols, patient characteristics, and lesion sets. Randomized data with longer-term follow-up would help validate these results [140].

A recent systematic review and meta-analysis of 15 randomized clinical trials involving 1219 patients found that sinus rhythm restoration was significantly higher in the MV repair and ablation group at discharge. The 1-year mortality was lower in the MV repair and ablation group (5.43% vs. 5.91%) [141].

### 5.3 Current Challenges and Future Perspectives

**Fig. 8.**
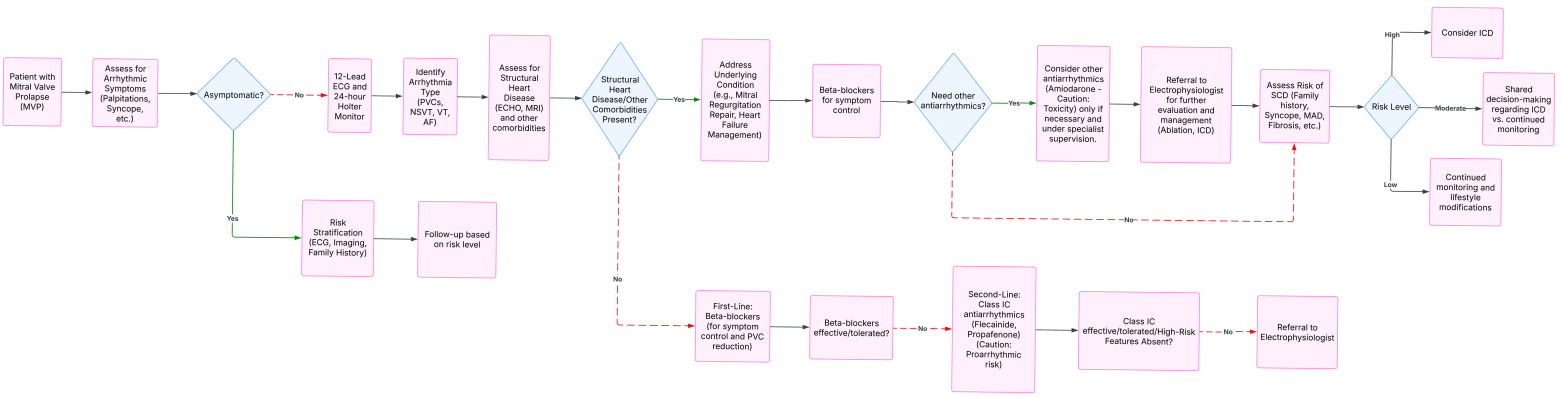
**MVP management algorithm**.

MVP is considered a benign condition; however, over time, the MV can undergo several changes that may require some degree of monitoring or treatment. The current perspective on MVP management, with or without arrhythmia, is presented in Fig. 8.

Surgery appears to be reserved for a small number of patients with MVP and MR. Considering the progress of cardiac surgery and the fact that the present lethality is close to 1%, elective surgery for AMVP due to severe degenerative MR should be reserved for patients who are likely to benefit from the procedure. The development of mini-invasive procedures, such as percutaneous repair of MV, may be an option in the future since this procedure is less invasive than major surgery. Current studies should focus on emerging imaging techniques, such as artificial intelligence (AI)-assisted diagnoses, which can enhance the early detection of arrhythmic risks in patients with MVP, as well as large-scale, prospective studies to validate the long-term benefits of surgical repair.

## 6. Conclusions

Current studies have demonstrated that MV surgery can be beneficial for patients with AMVP due to severe degenerative MR. A small subgroup of patients with AMVP and severe myxomatous disease, irrespective of degenerative MR, may also benefit from the procedure. Mini-invasive procedures, such as percutaneous repair of MV, might be an option for patients who cannot undergo major surgery.
